# Gangrene of Lungs

**Published:** 1914-11

**Authors:** 


**Gangrene of Lungs.** G. Guisez (Bull. de l'Acad. de Med.) injects from 20 to 25 cc. of medicated oil directly into the bronchus. In ten patients with gangrene of the lungs all recovered under this treatment. They had single or double gangrene, with fever and extreme prostration. The local processes healed up and the expectoration ceased. Others have confirmed these findings, but cases of tuberculosis are more refractory. Improvement, however, has been noted. Among other drugs used, Guisez employed a 5 or 10 per cent. solution of Guaiacol in oil.

